# Sudden weaning of angel fish *pterophyllum scalare* (Lichtenstein) (Pisces; Cichlidae) larvae from brine shrimp (*Artemia* sp) nauplii to formulated larval feed

**DOI:** 10.1186/2193-1801-2-102

**Published:** 2013-03-12

**Authors:** Sandamali Sakunthala Herath, Kerthi Sri Senarathna Atapaththu

**Affiliations:** 1 Department of Fisheries and Aquaculture, Faculty of Fisheries and Marine Sciences & Technology, University of Ruhuna, Matara, Sri Lanka; 2 Department of Limnology, Faculty of Fisheries and Marine Sciences & Technology, University of Ruhuna, Matara, Sri Lanka

**Keywords:** Angel fish, Larvae, Weaning, Artemia nauplii, Formulated feed

## Abstract

This study investigated the effects of sudden weaning of angel fish larvae (*Pteraphylum scalari*) from *Artemia* nauplii to commercial larval feed. Four days post hatch (DPH) larvae were reared in four different weaning protocols (TR1-TR4) with triplicates in a complete randomize design. Larvae in TR1 and TR4 were exclusively fed *Artemia* nauplii and dry feed respectively. In TR2 and TR3, larvae were initially fed *Artemia* nauplii and suddenly wean to formulated feed on 14 DPH and 7 DPH respectively. The experiment was lasted for 28 days. At the end of the experiment, final mean weight (FW), total length (FL), height (FH), Daily Weight Gain (DWG), Specific Growth Rate (SGR), survival and stress index were compared. Significantly highest (P < 0.05) FW, DWG and SGR were observed in TR1 and TR2 while former values of TR3 were not significantly different from TR1. Highest FL observed in TR1 and TR2 while FL of TR2 was statistically similar to that of TR3. The poorest growth was observed in larvae solely fed formulated feed. Survival and the stress index were independent from weaning methods. Although sudden weaning is possible on 7 DPH, larvae showed comparatively higher growth when switch off to formulate feed on 14 DPH.

## Introduction

Seed production and larval rearing are the most critical steps in the aquaculture production chain. Success of this stage is determined by a combination of factors including larval nutrition, environment, immunity, water quality and stocking density. Among them, feeding and nutrition of tiny fish larvae plays a significant role and live feed is generally considered as the most suitable feed for first feeding larvae (Conceição et al. 2010). *Artemia* nauplii, rotifers, *Moina*, blood worms, *Brachionus* and copepods are some of the commonly used live feeds in aquaculture. However, associated constraints of live feed production such as high cost, potential risk on disease transmission, required additional infrastructure and manpower create difficulties to hatchery operations (Faulk & Holt,^2009^ ; Person 1989). Further, some of the live feeds are needed to be enriched with nutrients before feeding as they lack some essential factors required to maintain optimum growth and survival of fish larvae (Sargent et al. 1997 ; Aragão et al. 2004). However, readily available dry formulated larval feeds have an advantage over live feeds as it is more convenient to use and less expensive. Therefore, weaning larvae onto dry formulated diets at their early stage provides economical and practical benefits to hatchery operations. Different weaning protocols with a varying degree of success have been reported in aquaculture literature for both freshwater and marine fish species (Hung et al. 2002 ; Bonaldo et al. 2011 ; Muguet et al. 2011 ; Wang et al. 2009 ; Ballagh et al. 2010).

Angel fish (*Pteraphylum scalari*) is one of the popular species among aquarium due to its shape, colour, ability to tolerate a wide range of environmental conditions and ease of spawning. Brine shrimp nauplii (*Artemia* sp) are widely used as an excellent starter feed for both freshwater and marine species and it is usually offered to angel fish larvae during the first 3–4 weeks (pers.com). *Artemia* is expensive and its supply varies with the environmental changes (Lavens & Sorgeloos 2000). Hence, shortening of *Artemia* feeding phase would provide both economical and practical benefits to hatchery operations and weaning techniques are yet to be developed for angels. Further, there is no any single study that has been undertaken to study the effect of sudden weaning of angel fish larvae from live feed to dry formulated feed. Therefore, this study was designed to evaluate the effects of sudden weaning from *Artemia* nauplii to formulated feed on growth and survival of angel fish (*Pterophyllum scalare*) larvae.

## Methodology

### Experimental fish and culture units

Angel brood fish were obtained from the aquarium of the Faculty of Fisheries and Marine Sciences and Technology (FMST), university of Ruhuna, Sri Lanka. Male and female brood fish were stocked in a glass tank prior to the commencing of the experiment. Two couples of angel fish were selected after coupling and transferred to breeding tanks for spawning. Four days post hatched (DPH) healthy larvae were used as experimental fish and glass tanks (30 × 20 × 20 cm) were used as experimental units. This research was approved by the Research Committee of the FMST. All experimental steps were conducted using the aquaculture research facilities of the FMST, University of Ruhuna, Sri Lanka.

### Experimental design

Four different weaning methods (TR1-TR4) each with three replicates were randomly allocated into 12 (4 × 3) experimental units in a Complete Randomize Design (CRD). In TR1 and TR4, larvae were exclusively fed *Artemia* nauplii and formulated feed respectively throughout the whole experiment. Larvae in TR2 and TR3 were initially fed with *Artemia* nauplii until 14 DPH and 7 DPH respectively and suddenly offered formulated feed (Table 1). All tanks were filled with de-chlorinated tap water up to 18 cm. We introduced larvae to the experimental tanks by using a deep spoon. Larvae were collected to the spoon from the stock tank and allow to slowly mixing water in experimental tank. Larvae were stocked in each tank at a stocking density of 30 larvae per tank and allow larvae to adjust to the new system for 48 hours before starting the experiment.

**Table 1:** **Number of days that live feed and formulated feed was offered at weaning methods employed**

	TR1	TR2	TR3	TR4
*Artemia* (days)	28	14	7	0
Formulated feed (days)	0	14	21	28
Total (days)	28	28	28	28

### Feeding protocol

*Artemia* nauplii were obtained after hatching *Artemia* cysts (Premier Cyst, Lanka Salt Ltd, Sri Lanka) in a 35 ppt saline solution. Commercial larval feed (Prima feed No 2, Prima Ceylon Ltd, Sri Lanka) was purchased from the local market and their composition is given in the Table 2. Dry feed was ground before feeding and larvae were fed to satiation twice a day at 08.00 h and 14.00 h. Water quality on experimental tanks was maintained by avoiding the accumulation of fish fecal matters and uneaten feeds. Every day in the morning, fish fecal matters and uneaten feeds were siphoned out before feeding and the water losses were compensated using de-chlorinated tap water. Daily mortality was recorded and the experiment was lasted for 28 days.

**Table 2:** **Nutrient data from the manufactures for the formulated feed**

	% composition
Protein	42
Lipid	10
Fat	4
Ash	12
Metheonine	0.94
Calcium	2.5
Phosphorous	1.2

### Stress test

The stress test entailed exposure of the fish larvae to an osmotic shock in a saline solution. A preliminary trial was conducted before performing the stress test according to Lim et al. (2002) to select the appropriate salinity level to be used in the subsequent stress resistance test. A series of saline solution (10, 20, 25, 30, 35 and 40 ppt) was prepared using sodium chloride and aerated tap water. Each concentration was considered as a treatment and this preliminary trial was conducted using glass beakers (1000 ml). Each treatment with two replicates were randomly allocated into 12 (6 × 2) beakers in a CRD. Beakers were filled with the relevant saline solution up to 500 ml mark and ten angel fry (28 days old) were stocked to each beaker. Mortality pattern was observed for 120 minutes and cumulative mortality was recorded at every five minutes.

After selecting the appropriate salinity on the basis of the former experiment (20 ppt), stress resistance test was conducted to evaluate stress resistance of fish larvae weaned at four different weaning techniques following the method described by Lim et al. (2002)*.* Ten larvae from each treatment (TR1-TR4) along with triplicates were exposed to an osmotic shock by putting larvae in a 20 ppt saline solution. Mortality pattern of fish larvae was observed for 120 minutes and cumulative mortality were recorded at every 3-min. Stress resistance was expressed as the stress index, calculated as the average sum of cumulative mortality value in triplicate beakers according to the former authors.

### Analytical procedure

At the commencement of the experiment, randomly selected 20 fish larvae were batch weighted to measure the initial mean weight (IW). Length of larvae (Total length) was individually measured using a stage micrometer and a light microscope. Larvae were not sampled during the study period to avoid stress. At the end of the experiment, eight larvae were randomly selected from each treatment to measure final mean weight (FW), final length and the height. Larvae were individually measured for weight using an analytical balance. Total length and height were measured using a measuring board.

Daily weight gain (DWG - mg day^-1^) = [FW – IW] / number of days; specific growth rate (SGR%) = (ln FW –ln IW) / number of days × 100; Survival rate (SR) (%) = Number of fish at the end of the experiment / number of fish stocked × 100, stress index were computed.

Data are presented as mean ± standard deviation (SD). Data analysis was done with SPSS (16 version) statistical software. Before being analyzed, data were checked for normality. Results were compared by analysis of variance (one way ANOVA) followed by the Tukey’s multiple range test when significant differences were found at the p = 0.05 level.

## Results

### Selection of suitable salinity for stress test

**Figure 1:**
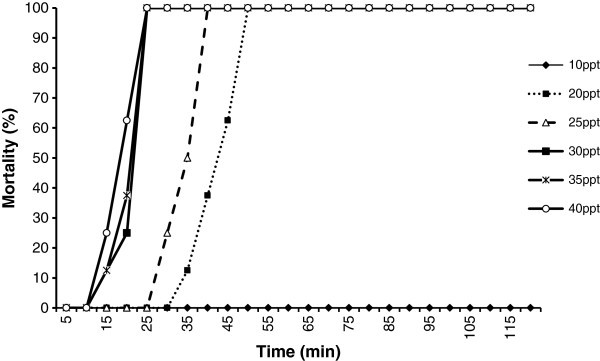
**Percentage mortality of fish larvae at different salinity levels.**

Mean weight and mean length of larvae used for the preliminary stress test were 30 mg and 13 mm respectively. Observed trend in larval mortalities over 120 minutes period at different salinities are presented in Figure 1.

In 40, 35 and 30 ppt salinities, 100% mortality observed within 20 minutes. But, all fish died in 25 and 20 ppt salinity levels after 40 minutes and 50 minutes respectively. However 100% survival was observed even at the end of the experiment (after 2 hours) when fish were exposed to 10 ppt salinity. Therefore, 20 ppt saline solution was selected as the optimum salinity level to be used in subsequent stress resistance tests.

### Growth and survival

Initial wet weight, total length and the height of fish larvae were 0.10 mg, 4.5 mm and 1 mm respectively. Growth performances observed in fish larvae after four weeks trial are presented in Table 3.

**Table 3:** **Growth performances and survival rate of angel fish larvae reared under four different weaning methods for 28 days (values are in mean ± Standard deviation, n =3)**

Responses	Treatments			
	TR1	TR2	TR3	TR4
Final weight (mg)	35.4 ± 3.4^ab^	40.5 ± 0.2^a^	28.6 ± 4.8^b^	15.2 ± 2.6^c^
Final length (mm)	14.4 ± 0.50^a^	13.9 ± 0.32^ab^	12.8 ± 0.70^b^	9.5 ± 0.58 ^c^
Final height (mm)	4.9 ± 0.2^a^	4.7 ± 0.2^ab^	3.5 ± 0.3^b^	2.5 ± 0.4 ^c^
DWG (mg day^-1^)	0.9 ± 0.2^ab^	1.1 ± 0.0^a^	0.7 ± 0.2^b^	0.2 ± 0.1^c^
SGR (%)	4.54 ± 0.33^ab^	5.03 ± 0.02^a^	3.75 ± 0.62^b^	1.50 ± 0.64^c^
Survival (%)	73 ± 14^a^	70 ± 18^a^	68 ± 6^a^	51 ± 14^a^
Stress Index	232 ± 90^a^	226 ± 14^a^	290 ± 22^a^	253 ± 52^a^

Different superscripts in the same raw indicate significant differences at P < 0.05. (TR1: exclusively fed *Artemia*, TR2: *Artemia* for fist 14 days and rest formulated feed, TR3: *Artemia* for fist 7 days and rest formulated feed, TR4: exclusively fed formulated feed).

Final mean weight, daily weight gain and specific growth rate of larvae were significantly higher (P < 0.05) in TR2 and TR1. However, former growth performances of TR1 are not significantly different either from TR2 or TR3. Except final mean length, growth parameters of TR1, TR2 and TR3 are approximately 1.5 times higher when compared to TR4.

**Figure 2:**
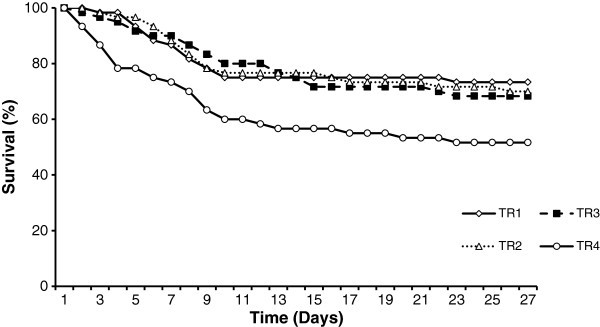
**Survival rate of fish larvae observed at four different weaning methods.**

Final mean length showed a somewhat different pattern than the final weight where the significantly highest length was observed in TR1 and TR2 while TR2 is not statistically different from TR3. Final height showed similar trend as observed for the length. Significantly least performances were observed in TR4 where the fish were solely fed formulated feed. Survival rate and the stress index were independent from the weaning method. Observed trend in larval survival over the entire experimental period is shown in Figure 2.

A comparatively high rate of mortality was observed during the first ten days in all treatment where the survival of larvae exclusively fed formulated feed decreased approximately to 60% (Figure 2). After 10 days, survival of each treatment did not fluctuate in a wide range.

**Figure 3:**
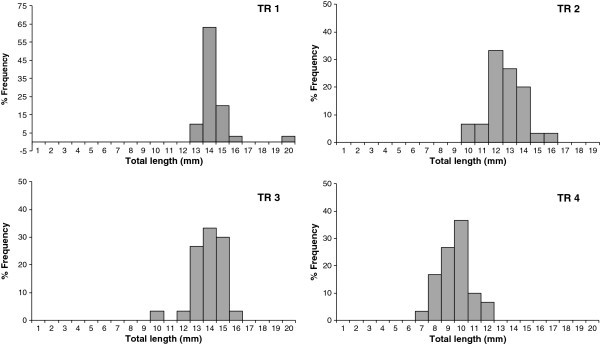
**Total length frequency histograms of angel fish larvae at day 28 in TR1: exclusively fed*Artemia*nauplii, TR2: first 14 days*Artemia*nauplii and rest is formulated feed, TR3: first 7 days*Arteia*nauplii and rest is formulated feed, TR4: exclusively fed dry feed (class interval: 1 mm, values are means of triplicates).**

Length class distributions of larvae at the end of the trial are presented in Figure 3. Total length of larvae reared in TR1, TR2, TR3 and TR4 ranged in 13–20 mm, 10–16 mm, 10–14 mm and 7–12 mm respectively.

The length frequency distributions of all treatments approximately followed a normal distribution pattern. Analysis of length frequency histograms demonstrated continuous dry feed feeding (TR4) yields appreciable percentage of smaller larvae than other treatments. Mean length of TR4 (9.5 ± 0.58 mm) is always less than the smallest length classes of other treatments.

## Discussion

The maximum time gap between first and last mortality in the preliminary study was observed in 20 ppt salinity and similar salinity ranges have been observed for some other freshwater species such as molly (35 ppt), swordtail fry (20 ppt), tetra fry ( (25 ppt) (Merchie et al. 15 ppt) and *Clarias gariepinus*^1995^; Lim et al. 2002). However, Garcia-Ulloa and Gomez-Romero (2005 ) used 30 ppt saline solution to measure the stress resistance of juvenile angels. The optimum salinities for such a test could vary even within the same species as stress resistance depends upon the age and the development stage of the larvae. However, Lim *et al.*^2002^) reported that the maximum time gap between the first and the last fish mortality in a stress test range from 40 minutes for platy to 75 minutes for molly fry. The observed time gap in present study (50 minutes) agrees with this range. As there is no specific stress resistance test for angel fish larvae, further studies focusing on different salinity levels under various exposure times are needed to develop a protocol to evaluate the stress resistance of angel fish larvae.

The present study observed that applied weaning methods did not significantly contribute for the stress resistance of fish larvae. Garcia-Ulloa and Gomez-Romero (2005) also conclude that stress index for angel fish juveniles is independent from type of feed offered after testing four different feeds viz; de-capsulated *Artemia* (, commercial flakes, commercial pellets and commercial starter diet. Lim *et al.*^2002^) reported that stress resistance of fish larvae is a function of sub optimal physiological condition of fish larvae that affected by infectious diseases, poor water quality and nutritional conditions. However, infectious diseases were not observed over the experimental period. Stress resistance index of platy, sword tail, molly and guppy after feeding with *Artemia* nauplii for 4 weeks were 180 ±37.59, 114.0 ± 7.4, 143.5 ± 21.79 and 233.5 ± 17.75 respectively (Lim et al. 2002). Present experiment was also conducted for the same period similar to the former study. We observed that stress index of larvae exclusively fed *Artemia* ( nauplii was approximately similar to that of guppies observed by Lim *et al.*^2002^). However, it was lower than that of molly, sword tail and platy.

Ribeiro et al. (2012) observed angel fish larval performances are independent from the feeding frequency. Further, similar growth performance were obtained by feeding angel fish larvae at four meals per day and two meals per day by l (Kasiri et al. 2011). On the other hand, two times per day frequency is more practicable and convenient to employ according to our experience. Present study revealed that, angel fish larvae are able to accept both live feed and formulated dry feed at the onset of feeding. However, larvae solely fed live feed showed comparatively higher growth performances than that of larvae exclusively fed formulated dry feed. Similar observation has also been reported for *Danio rerio* (Carvalho et al. 2006), *Heterobranchus. longifillis*(Kerdchuen & Legendre 1994), southern flounder, *Paralichthys lethostigma* (Faulk & Holt 2009), haddock (*Melanogrammus aeglefinus*) (Blair et al. 2003) and Whishker cat fish (*Macronema bleekeri)* (Dan 2008). Well developed digestive system with functional digestive enzymes are absent at the onset of feeding and exogenous enzymes from live feeds play an important role in digestion (Person 1989 ; Dbrowski 1982). Therefore, the least growth performances observed in larvae exclusively fed dry feed (TR4) could be attributed to a less developed digestive system which cannot support fish larvae to fully digest artificial dry feed. Further, *Artemia* nauplii contain nearly 56% protein and 17% lipid (Garcí a-Ortega et al. 1998 ; Nguyen et al. 2011) that make it as an excellent feed for first-feeding larvae. Aquaculture organisms are in need of ascorbic acid (vitamin C) during their developmental stages and the requirements vary with the developmental stage. Different ascorbic acid derivatives have been observed in *Artemia* and the evidences are available in the literature for the liberation of vitamin C from the freshly hatched naupli (Dabrowski 1991 ; Nelis et al. 1994). On the other hand, *Artemia* can be pre enriched with nutrients. It was observed that fish larvae promptly captured and ingested *Artemia* nauplii than dry feed particles. It was further indicated by the orange coloured larval digestive tract a few minutes after feeding. Low ingestion rate of dry feeds and leaching nutrients from dry feeds have also been highlighted as factors behind the poor growth in fish larvae fed dry feeds (Muguet et al. 2011). Moreover, physical properties of feeds (Sarkar et al. 2006), developmental stage specific nutritional requirement of larvae are also responsible for the poor growth of larvae exclusively fed formulated feeds (Flüchter 1982).

Best growth performances were observed in both TR1 and TR2 followed by TR3 and TR4. Nevertheless, growth performances of larvae exclusively fed *Artemia* was not significantly different from TR2, suggesting that angel fish larvae are possible to wean from 14th DPH without affecting growth of larvae. Therefore, switching larvae to dry feed on the 14th day after feeding with *Atemia* nauplii is the best way to produce angel fish larvae than exclusively feed *Artemia* over the whole larval production cycle. However, it is necessary to provide *Artemia* (nauplii at least for the first seven or more days in order to obtain acceptable growth performances. Hung *et al.*^2002^) reported bassa catfish (*P. bocourti*) can also be weaned to trout pellets after feeding *Artemia* nauplii for the first three or more days and then be fed with artificial feeds. Although growth performances were significantly low (P < 0.05) in larvae exclusively fed dry feeds, angel fish accepted formulated feed even at the first feeding without any additional weaning protocol. A similar trend has also been reported by Ljunggren *et al.*(2003) for pike perch juveniles. Further, Carvalho *et al.*^2006^) also observed significant growth and survival (56%) in zebra fish larvae exclusively fed dry feeds. Present study agrees with the former survival rate as 51% survival observed in TR4 where larvae were solely on formulated feed. However, Sales (2011) reported that larvae solely fed formulated diets have a 2.5 times higher chance to die than those who fed live feeds. But our findings contradict with former observation as an acceptable survival (51%) observed in larvae exclusively fed dry feeds compared to other treatments. On the other hand, statistical analysis revealed that, larval survival was independent from the weaning methods employed. Although eight larvae per replicate were sampled for the final measurements, less number of fish was measurement only in some replicates of TR4 due to high mortality. In such a condition, all remaining larvae after using for the stress test were measured to calculate the mean weight, length and the height.

Some studies employed co-feeding where both live feed and dry feed offer at the same time (Nhu et al. 2010 ; Wang et al. 2009) and live feed portion is gradually decreased by increasing the dry feed portion. As co-feeding was not tested in the present study, further steps are needed to compare the effects of sudden weaning and co-feeding. Possibility of direct weaning from live feed to formulated feed was tested in this by selecting a commercially available formulated feed. This feed was selected as it is one of the most popular commercial diets in the local market and widely used by local farmers (Pers.com). Further, it also contained acceptable protein content (42%). Although, present study revealed that the possibility of sudden weaning of angel fish larvae, nutritional composition of larval feed particularly amino acid and fatty acid composition, and stocking density of larvae are needed to be considered to develop a complete weaning protocol.

## Conclusion

Results of the present study indicate the possibility of sudden weaning angel larva *P.Scalari* from brine shrimp to dry formulated feed at their early life stages. Although, angel fish larvae show an appreciable growth and survival after shifting to dry feed on 7 DHP, it would be more beneficial to wean suddenly on 14 DPH. This reduces the dependence on expensive brine shrimp, makes weaning easy and would imply a hatchery cost reduction.
